# G-quadruplexes and G-quadruplex ligands: targets and tools in antiviral therapy

**DOI:** 10.1093/nar/gky187

**Published:** 2018-03-15

**Authors:** Emanuela Ruggiero, Sara N Richter

**Affiliations:** Department of Molecular Medicine, University of Padua, Padua 35121, Italy

## Abstract

G-quadruplexes (G4s) are non-canonical nucleic acids secondary structures that form within guanine-rich strands of regulatory genomic regions. G4s have been extensively described in the human genome, especially in telomeres and oncogene promoters; in recent years the presence of G4s in viruses has attracted increasing interest. Indeed, G4s have been reported in several viruses, including those involved in recent epidemics, such as the Zika and Ebola viruses. Viral G4s are usually located in regulatory regions of the genome and implicated in the control of key viral processes; in some cases, they have been involved also in viral latency. In this context, G4 ligands have been developed and tested both as tools to study the complexity of G4-mediated mechanisms in the viral life cycle, and as therapeutic agents. In general, G4 ligands showed promising antiviral activity, with G4-mediated mechanisms of action both at the genome and transcript level. This review aims to provide an updated close-up of the literature on G4s in viruses. The current state of the art of G4 ligands in antiviral research is also reported, with particular focus on the structural and physicochemical requirements for optimal biological activity. The achievements and the to-dos in the field are discussed.

## INTRODUCTION

**Figure 1.**
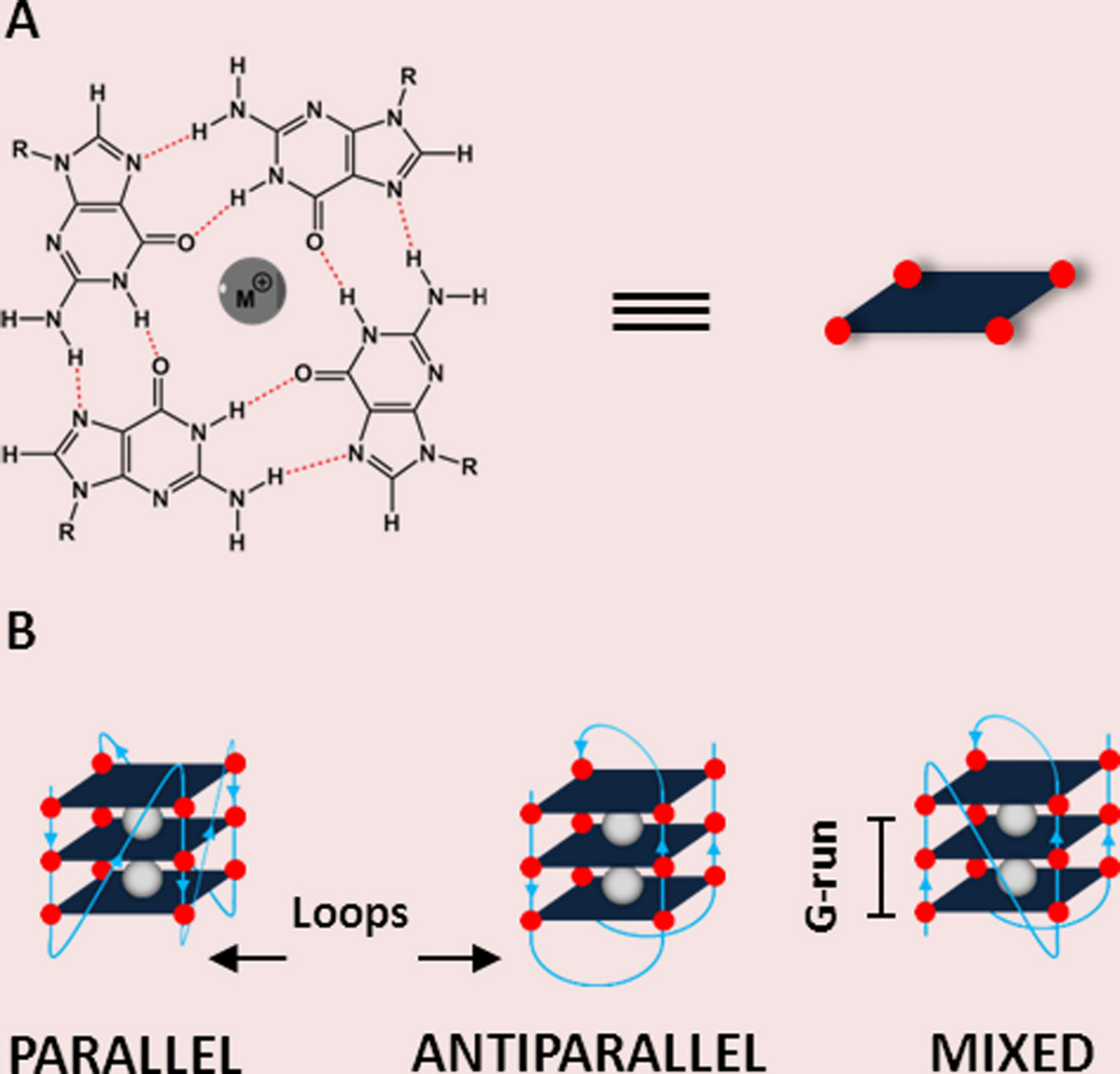
The G-quadruplex structure. (**A**) Chemical structure (left) and schematic illustration (right) of a G-tetrad, composed of four guanines linked together through Hoogsteen H-bonds (red dashed lines); M^+^ represents the monovalent cation coordinated at the center of the tetrad. (**B**) Different topologies of intramolecular G4 structures.

G-quadruplexes (G4s) are nucleic acids secondary structures that can form within DNA (1) or RNA (2) guanine (G)-rich strands, when two or more G-tetrads stack on top of each other and coordinate monovalent cations, such as K^+^ and Na^+^. Each tetrad is composed of four G residues that are linked by the sugar–phosphate backbone and connected through Hoogsteen-type hydrogen bonds. G4s are highly polymorphic structures whose topology can be influenced by variations in strand stoichiometry and polarity, as well as by the nature and length of loops and their location in the sequence. G4s can fold intramolecularly from a single G-rich strand, or intermolecularly through dimerization or tetramerization of separate filaments: research of biologically relevant G4s has mainly focused on monomolecular G4s (3,4); however, intermolecular G4s are gaining increasing attention (5–7). Strands orientation defines the parallel, antiparallel or mixed topology of G4s, which is directly correlated to the conformational state, *anti* or *syn*, of the glycosidic bond between the G base and the sugar (1). The *anti* conformation characterizes a parallel folding, while antiparallel G4s are found to adopt both *syn* and *anti* orientations (8) (Figure 1). While RNA G4s are mostly locked in a parallel conformation due to the 2′-hydroxyl group in the sugar which exclusively allows the *anti* orientation (2), DNA G4s are in principle characterized by higher topological diversity, even though the majority of DNA G4s examined so far adopt the parallel topology.

Computational analysis using different algorithms (9,10) indicated that 300 000 and up to around 3 000 000 potential G4-forming sequences may form in the human genome, correlated with specific gene functions (11). These data have been corroborated by ‘G4-seq’ high-throughput sequencing method, which identified about 700 000 G4s (12). However, mapping of G4s in chromatin by G4 ChIP-sequencing with an anti-G4 antibody (13) or footprinting (14) retrieved only about 10 000 G4s in highly transcribed regulatory nucleosome-depleted chromatin regions. These data indicate that G4s are mostly suppressed in chromatin and that, in turn, they may influence the occupancy and positioning of nucleosomes.

In general, G4 sequences are non-randomly distributed but mainly clustered in pivotal genomic regions, namely telomeres, gene promoters and DNA replication origins (15). Moreover, putative G4-forming sequences have been found in coding and non-coding regions of the human transcriptome, i.e. open reading frames and untranslated regions (UTRs), and in the telomeric repeat-containing RNA (2).This evidence suggests that G4s are likely involved in the regulation of different biological pathways such as replication, transcription, translation and genome instability.

In the past years, the resolution of G4 structures (16–18) and the employment of novel visualization approaches (19–21) helped researchers to validate the previous computational predictions, disclosing new aspects of the multi-faceted G4s world, e.g. the effective occurrence of G4s within patient-derived cancer tissues (22) or the key role in the pathogenesis of two incurable neurodegenerative diseases, amyotrophic lateral sclerosis and frontotemporal dementia (23). Indeed, the presence of G4s in the human genome and their potential in diseases modulation have been extensively investigated, resulting in many good and exhaustive reviews focused on G4 structures (1,8,24,25) and their biological role, particularly in telomeres (26–29) and oncogene promoters (30–35).

## G-QUADRUPLEXES IN VIRUSES: PRESENCE AND FUNCTION

Besides humans, putative G4-forming sequences have been found in other mammalian genomes (36), yeasts (37), protozoa (38), bacteria (39,40) and viruses, therefore implicating G4s in many human infectious diseases. One review has been published in 2015 on the possible role of G4s in the antigenic variation systems of bacteria and protozoa and silencing of two viruses (41). The possible role of G4s in viruses and the use of G4-forming oligonucleotides as antiviral agents have been discussed in 2014 (42).

**Figure 2.**
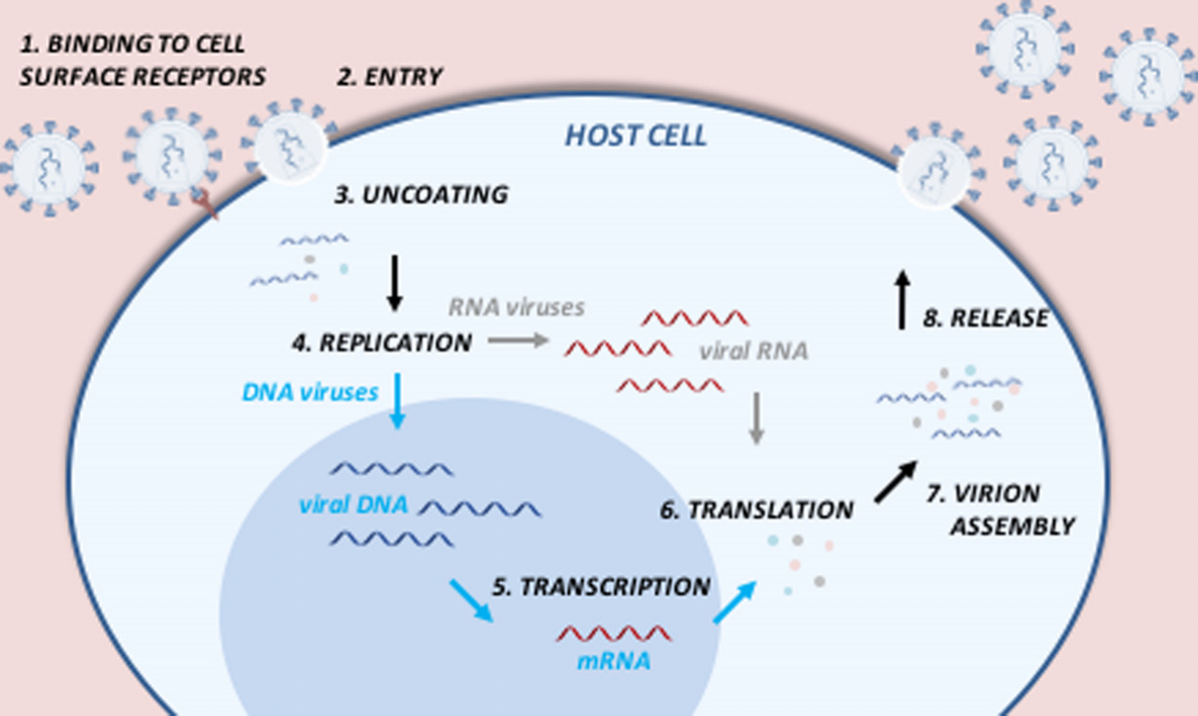
Schematic representation of the viral life cycle. The virus recognizes and binds the host cell surface receptors (step 1) to enter the cell (step 2). After penetration, the viral genome is uncoated (step 3) and its DNA or RNA nature determines where and how the genome is replicated (step 4): most DNA viruses replicate in the cell nucleus, while the majority of RNA viruses replicate in the cytoplasm of infected cells. After viral mRNA production, viral proteins are expressed in the cytoplasm (steps 5–6). The newly synthesized viral genomes and proteins are then assembled into new virions (step 7), which are released outside the cell (step 8).

**Figure 3.**
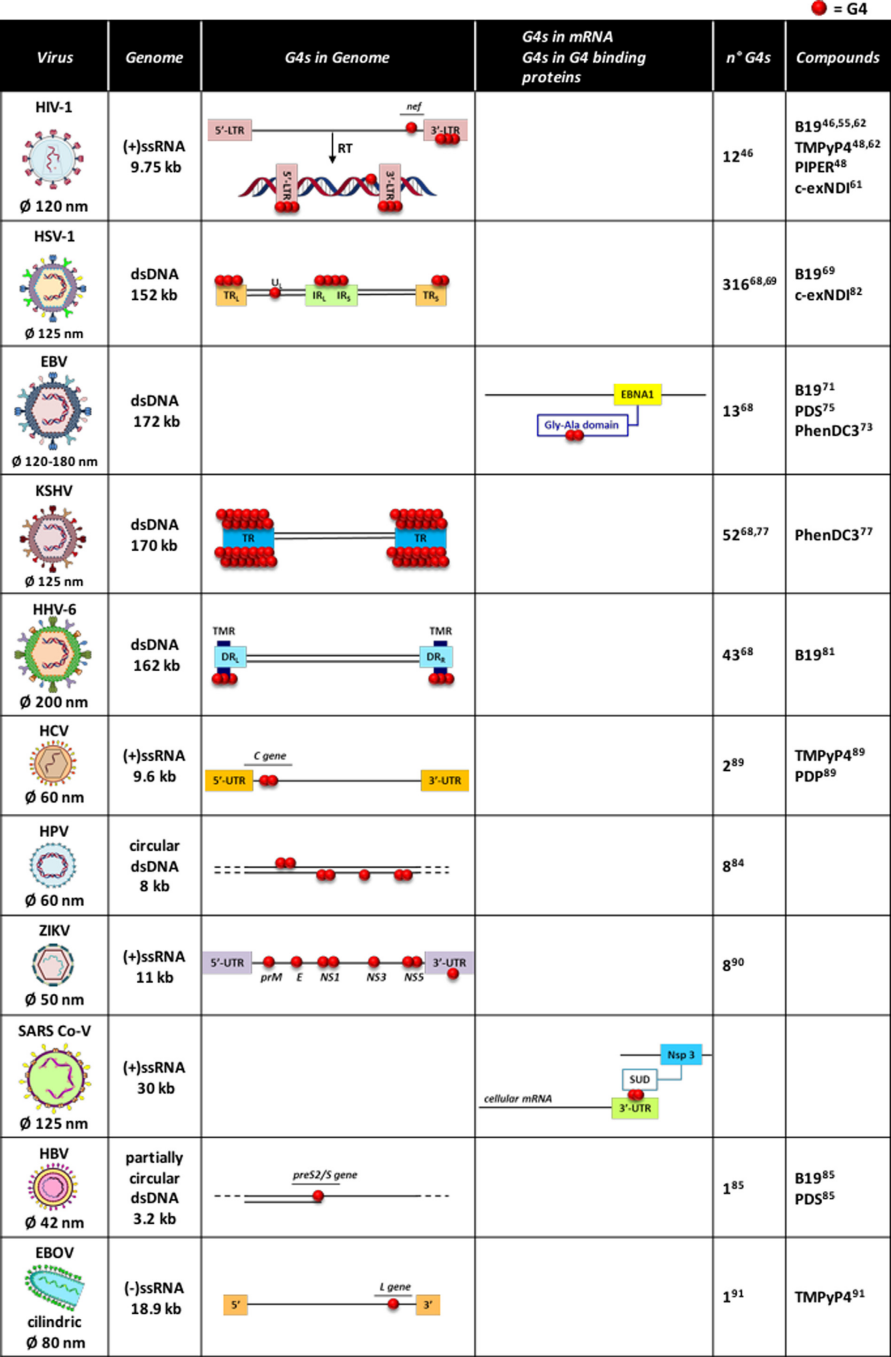
Summary of G4s reported in viral genomes. For each virus the following information is shown: virion structure and dimension, genome size and organization; schematic representation of the G4 (red dots) location in the viral genomes or in the mRNA and G4 binding proteins; number of G4s assessed through bioinformatics analysis, according to the corresponding references; G4 ligands reported to date to display antiviral effect and corresponding references.

Since the number of reports describing the presence of G4s in virus genomes has boomed in the past 2 years and treatment with several G4 ligands has shown potentially interesting therapeutic activity, we here aim at presenting, organizing and discussing an up-to-date close-up of the literature on G4s in viruses and the classes of molecules that have shown antiviral activity by viral G4 targeting. In particular, we first focus on the presence and proposed function of G4s in virus genomes. Next, we present the classes of G4 ligands that have reported successful antiviral activity, with special emphasis on the structural and physicochemical properties that characterize the viral G4/G4 ligand interaction. A general simplified virus life cycle is schematically depicted in Figure 2; a summary of the viruses in which G4s have been reported and of the corresponding G4s is shown in Figure 3. Since the use of G4-forming oligonucleotides as antiviral agents has been more recently addressed by Musumeci *et al.* (43,44), this topic has not been considered in the present review.

### Human immunodeficiency virus

The human immunodeficiency virus (HIV) is the etiological agent of the acquired immune deficiency syndrome (AIDS), which to date affects more than 35 million people worldwide. Albeit the current anti-retroviral therapy keeps the disease progression under control, people still die from HIV-related causes; thereby it is necessary to find alternative and effective antiviral targets. The HIV belongs to the *Retroviridae* family; the single-stranded RNA genome is processed by the viral retrotranscriptase and the newly formed double-stranded DNA is integrated into the host cell chromosomes to form the proviral genome, from which viral mRNAs and new genomes are transcribed.

The research of G4s in the HIV-1 genome has been quite productive, concerning not only the two RNA viral genome copies, but also the integrated proviral genome, specifically in the long terminal promoter (LTR) region (45–47) and in the *nef* coding region (48), as properly reviewed by Metifiot *et al.* (42).

Briefly, the LTR promoter is characterized by a highly conserved G-rich sequence in the U3 region, corresponding to Sp1 and NF-κB binding sites, where three mutually exclusive G4 structures can form, i.e. LTR-II, LTR-III and LTR-IV (46). LTR-IV is a parallel G4 with a bulge at its 3′-end, as ascertained by nuclear magnetic resonance (NMR) characterization (49). LTR-III and LTR-IV exert opposite effects on LTR promoter activity, which is silenced when LTR-III is folded and enhanced by LTR-IV stabilization (49). In addition, the LTR G4 region is under the control of two nuclear proteins: nucleolin, which upon binding increases LTR G4 stability and thus silences transcription (50) and the human ribonucleoprotein (hnRNP) A2/B1, which unwinds the LTR region, decreasing its promoter activity (51): these data suggest that the balance between G4s acts as a regulatory mechanism in HIV-1 promoter activity. Interestingly, G4-forming sequences are present in the LTR promoter of all primate lentiviruses and display binding sites for transcription factors that are related to G4 regulation (52,53), supporting a role for G4s as crucial control elements for viral transcription, conserved throughout evolution (54).

G4s were also evidenced in the U3 region of the HIV-1 RNA genome, where multiple highly stable parallel G4s can form (55). RNA sequences can dimerize through an intermolecular G4 interaction (56), suggesting that the U3 region could represent an additional point of contact between the two viral genome copies. Additionally, such RNA G4s likely contribute to the observed increased genetic recombination rate in the U3 (57).

*Nef*, a viral accessory protein, is an essential factor in proviral DNA synthesis (58) and in the establishment of a persistent state of infection (59). Its coding region is located at the 3′-end of the viral genome and partially overlaps with the 3′-LTR. Three G4 sequences have been identified in the most conserved region of the gene (48).

G-rich sequences able to form G4s were reported in the HIV central DNA flap overlapping positive-strand and were found to protect the pre-integrated genome from nuclease degradation (60).

Stabilization of HIV G4s by small molecules showed antiviral effects at different levels: G4 ligand binding to DNA LTR G4s decreased viral transcription, while binding to RNA LTR G4s inhibited the reverse transcription process, leading in both cases to strong antiviral effects (46,55,61). G4 ligand-mediated stabilization of the *nef* G4s induced *nef*-dependent antiviral activity (48). Very recently, G4 stabilizing agents were also employed in cells infected with latent HIV-1, where their activity resulted in a strong antiviral effect, especially in combination with a DNA repair inhibitor, revealing new aspects of HIV-1 latent infection (62).

The specific molecules that were used as anti-HIV-1 agents are discussed in the ‘Antiviral G4 ligands’ section of this review.

### Herpesviruses

*Herpesviridae* is a large family of viruses with long linear double-stranded DNA genomes. Among the nine herpesvirus species that can infect humans, at least five are extremely widespread, i.e. herpes simplex virus 1 and 2 (HSV-1 and HSV-2), varicella zoster virus, Epstein–Barr virus (EBV) and cytomegalovirus, which cause orolabial and genital herpes (63), chickenpox and shingles (64), mononucleosis (65) and some cancers (66). More than 90% of adults have been infected with at least one of these (63). Herpesviruses also tend to display latent, recurring infections, with the virus remaining in some part of the infected organism and typically maintaining its genome as extrachromosomal nuclear episome (67).

Recent genome-wide bioinformatics analysis revealed an impressively high density of putative G4-forming sequences in all herpesvirus species (68). Indeed, the presence of G4s has been experimentally reported for HSV-1, EBV, Kaposi’s sarcoma associated herpesvirus (KSHV) and human herpesvirus 6 (HHV-6).

HSV-1 establishes life-long persistent infections with a viral lifecycle that involves latency and reactivation/lytic replication. More than half of the world population suffers from HSV infections, the outcome of which may become severe in immunocompromised patients. Anti-HSV-1 therapy can be very effective; however, the emergence of drug-resistant viral strains urges the discovery of anti-herpetic drugs with innovative mechanisms of action. The HSV-1 genome, characterized by 68% GC-content, was found to contain numerous and highly stable G4-forming sequences that are mainly located in the repeated regions (69). These HSV-1 G4s, visualized through a G4-specific antibody in infected cells at different time points post-infections, were shown to form in a virus cycle-dependent fashion: viral G4s form massively in the cell nucleus during viral replication, and localize in different cell compartments according to the viral genome movements (70).

EBV is associated not only with the well-known infectious mononucleosis, but also with a wider spectrum of illnesses, including several lymphoid malignancies. Studies on the presence and role of G4s in EBV proved that the genome maintenance protein EBV-encoded nuclear antigen-1 (EBNA1) stimulates viral DNA replication by recruiting the cellular origin replication complex through an interaction with RNA G4s (71). The EBNA1 mRNA itself is rich in G clusters able to fold into parallel G4s, which behave as *cis*-acting regulators of viral mRNA translation, producing ribosome dissociation. G4s in EBNA1 mRNA have been shown to modulate the endogenous presentation of EBNA1-specific CD8^+^ T-cell epitopes, which are involved in persistent infections (72). The cellular protein nucleolin counteracts this mechanism by interacting with EBNA1 mRNA G4s and thus downregulating EBNA1 protein expression and antigen presentation (73,74). G4s can also be observed in the mRNAs of other genome maintenance proteins that are known to regulate their self-synthesis, suggesting that G4s are exploited as structural regulatory elements by the virus (75).

KSHV is the etiological agent of all forms of Kaposi’s sarcoma and other numerous lymphoproliferative disorders, which mostly concern AIDS patients, and at the moment, no treatments for the lytic or latent infections are available (76). The KSHV genome is organized in a 137 kb long unique region, flanked by the terminal repeats, which are rich in G residues and able to form stable G4s, both in the forward and reverse strands (77).

HHV-6 is a ubiquitous virus that infects almost 100% of the human population. The diseases associated with HHV-6 include the febrile illness roseola infantum, also known as the sixth childhood eruptive disease (78). Reactivation of HHV-6 in immunosuppressed individuals is associated with adverse clinical outcomes, comprising life-threatening encephalitis or graft rejection in transplant patients (79). The HHV-6 genome presents telomeric regions at its termini, which can integrate into the telomeres of human chromosomes: integration is considered one possible mode of latency (80). Since telomeres can fold into G4s, these structures may be involved in the mechanism of HHV-6 integration. Indeed, stabilization of telomeric G4s by a G4 ligand inhibited HHV-6 chromosomal integration (81).

Stabilization of herpesvirus G4s by G4 ligands led to antiviral activity. In HSV-1, inhibition of DNA replication and reduction of late viral transcripts were observed (69,82). In EBV, a G4 ligand inhibited EBNA1-dependent stimulation of viral DNA replication (71) and EBNA1 synthesis (75). In contrast, another G4 ligand reduced nucleolin binding to EBNA1 mRNA (75), which in turn resulted in enhanced EBNA1 synthesis and antigen presentation (73,74).

Treatment of latently infected cells with G4 stabilizing compounds proved to negatively regulate viral replication, leading to a reduction in the KSHV genome copies (77).

G4 ligands used against herpesviruses are discussed in the ‘Antiviral G4 ligands’ section of this review.

### Other viruses

#### DNA viruses

The human papillomavirus (HPV) is a double-stranded DNA virus that can cause skin and genital warts and some types of cancer. Its genome displays several G-rich sequences: stable G4s form in only eight out of 120 identified HPV types; however, the G4-forming HPVs include some of the most high risk HPV types, responsible for the majority of cases of cervical cancer (83,84).

The Hepatitis B virus (HBV) is a partially double-stranded DNA virus, the best known member of the *Hepadnaviridae* family. It causes the hepatitis B disease, which may lead to cirrhosis and hepatocellular carcinoma. A single putative G4-forming sequence was discovered in the promoter region of the preS2/S gene in HBV genotype B and was found to fold into an intramolecular hybrid G4 structure. Surprisingly, the G4 acted as a positive regulator of HBV transcription, as revealed by luciferase reporter assays (85).

Adeno-associated viruses (AAV) are single-stranded DNA viruses of the *Parvoviridae* family. AAV are not currently linked to human diseases and have been used as delivery vectors for gene therapy. A recent study reported the presence of G4s in the AAV genome. The DNA binding protein nucleophosmin (NPM1), which is known to enhance AAV infectivity, directly interacts with G4s: 18 putative G4s were identified, located within the inverted terminal repeat region (86).

#### RNA viruses

Amongst RNA viruses, G4 putative sequences have been identified in three positive and single-stranded ones, namely the severe acute respiratory syndrome coronavirus (SARS-CoV), the Hepatitis C virus (HCV) and the Zika virus (ZIKV).

The SARS-CoV belongs to the family of *Coronaviridae*; its genome is about 29.7 Kb, which is one of the largest among RNA viruses. It has been identified after a massive outbreak in 2003 and is considered one of the most pathogenic coronaviruses in humans. Within the non-structural protein 3, the so-called SARS unique domain (SUD), which plays an essential role in viral replication and transcription, was found to preferentially bind G4-forming oligonucleotides (87,88). These may be found in the 3′-non-translated regions of mRNAs coding for host-cell proteins involved in apoptosis or signal transduction; therefore, it has been proposed that SUD/G4 interaction may be involved in controlling the host cell’s response to the viral infection.

The HCV belongs to the *Flaviviridae* family; it can cause both acute and chronic hepatitis, possibly leading to cirrhosis and liver cancer. Bioinformatics and biophysical analysis demonstrated the existence of two highly conserved G4 sequences in the C gene of HCV (89).

The ZIKV is also included in the family of *Flaviviridae*. It is transmitted to humans by mosquito bites; while in an adult it may cause mild symptoms or even be symptomless, it may be devastating in a pregnant woman as it causes microcephaly in the unborn child. Several G4 sequences were discovered in the positive strand of the ZIKV genome: 7 of these are conserved within more than 50 flavivirus genomes, suggesting an important role in the life cycle of these viruses. Furthermore, ZIKV presents an additional G4 in the unique 3′-UTR region, crucial for initial viral replication of the negative-sense strand (90).

Finally, G4s have been investigated in the Ebola virus (EBOV) and Marburg virus (MARV), two negative and single-stranded RNA viruses belonging to the *Filoviridae* family. These are deadly pathogens that cause haemorrhagic fever in humans and primates (91). The presence of G4 sequences in the negative strand of EBOV and MARV was assessed by a fluorescent probe (92).

Both ZIKV and EBOLV went through massive outbreaks in the past three years, which makes them two of the most dangerous agents of viral epidemics of the current decade.

In Figure 3, all the viruses in which G4s have been investigated are displayed. The stabilizing G4 ligands tested in some of these viruses are thoroughly described in the section below.

## ANTIVIRAL G4 LIGANDS: DEVELOPMENT, ANTIVIRAL ACTIVITY AND MECHANISM

In the past few years much effort has been directed toward the design of small molecules able to target G4s, leading to very promising potential therapeutics, especially against cancer. Several updated reviews describe the use of G4 ligands that target telomeres and oncogenes to treat cancer (8,34,93–95).

Despite the considerable achievements in antiviral research, viral infections still represent a major global threat for human health, causing significant morbidity and mortality. The recurrent onset of drug-resistant pathogens, combined with the fact that the majority of viruses still lack a specific vaccine, urges the development of novel therapeutic approaches for the management of viral diseases. To this end, G4 ligands provide both compounds with an innovative mechanism of action in antiviral treatment and valuable tools to better understand virus mechanisms.

In the section below G4 ligands reported to exert antiviral activity have been grouped based on the chemical nature of their core. A description of their discovery, general G4 binding activity and biological effects in cells is initially provided. Antiviral properties, activity and selectivity are then discussed.

### BRACO-19

**Figure 4.**
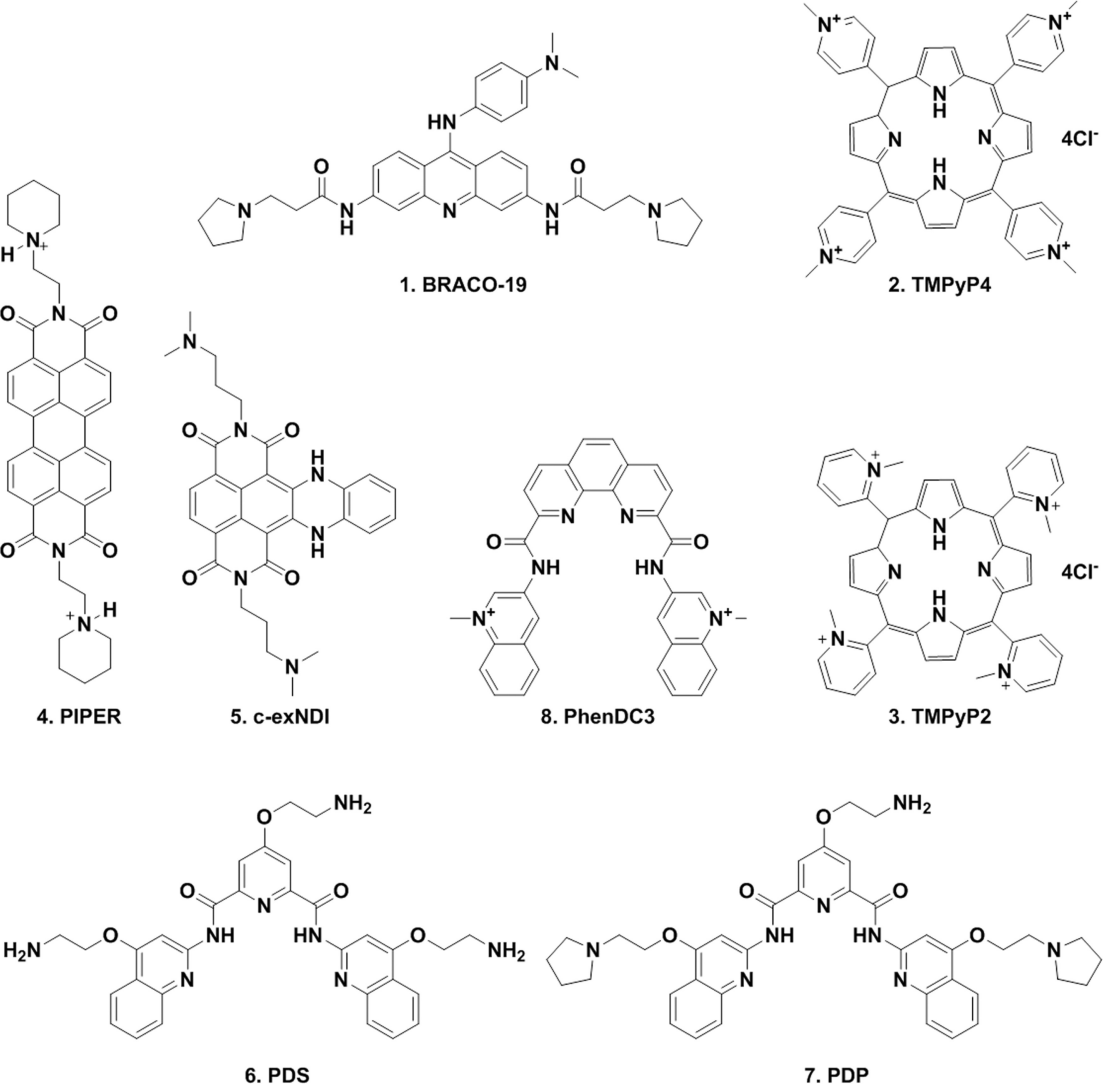
Chemical structures of reported G4 ligands with antiviral activity.

**Figure 5.**
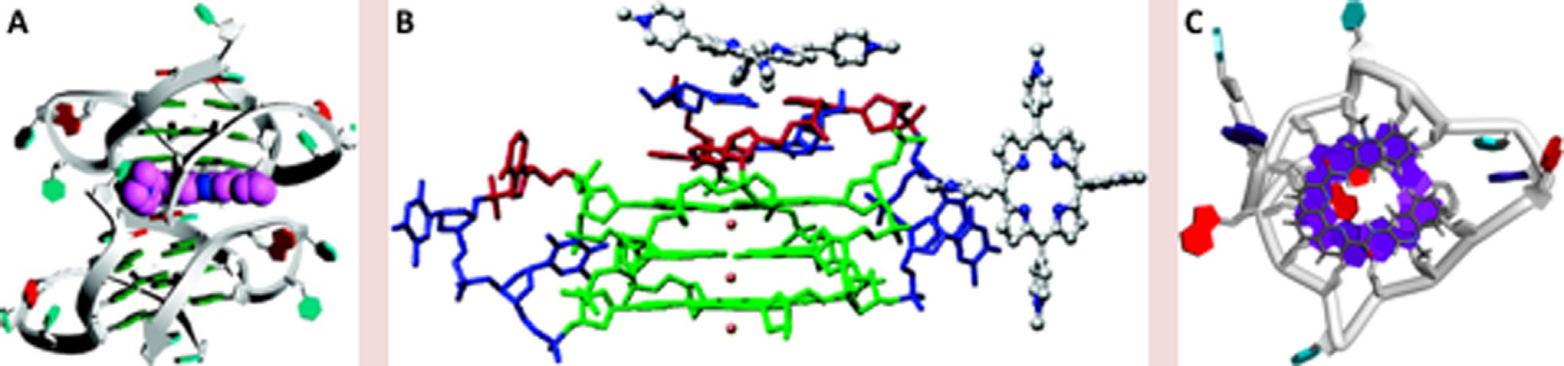
Solved crystal structures for three of the compounds discussed in the text. (**B**) Crystal structure of the complex between B19 and the bimolecular human telomeric G4 (PDB ID: 3CE5): each quadruplex contains three planar stacked G-tetrads with the molecule stacking directly onto the 3′ end quartet (136). (**B**) Crystal structure of the complex between TMPyP4 and the bimolecular human telomeric G4 (PDB ID: 2HRI). The compound binds by stacking onto the TTA nucleotides, as part of the external loop or at the 5′ region of the stacked quadruplex (105). (**C**) Crystal structure of the complex between PhenDC3 and the human c-myc-promoter G4 (PDB ID: 2MGN). The ligand establishes an optimal interaction with the top G-tetrad, while the two *N*-methyl groups are positioned above the grooves and have minimal contact with the tetraplex (132).

The *N,N'*-(9-((4-(dimethylamino)phenyl)amino)acridine-3,6-diyl)bis(3-(pyrrolidin-1-yl)propan-amide), labeled BRACO-19 (B19) (**1**, Figure 4), is to date one of the most studied G4 ligands. It is the outcome of a complex and thorough medicinal chemistry investigation that started with the introduction of an acridine moiety as a new chromophore in the research of G4 binders. Read and colleagues demonstrated that the acridine core was more active than the previously developed anthraquinone core (96,97), because of the presence of a nitrogen atom in the heterocyclic scaffold that could be protonated at physiological conditions. As a result, the electron deficiency in the chromophore was increased, with consequent enhancement of the G4 interaction (98). In-depth structure-activity relationship (SAR) analysis supported by molecular modeling techniques next led to the development of bi- and tri-substituted derivatives (99,100). These classes of compounds are characterized by a central planar pharmacophore that binds G-tetrads through π–π interactions (Figure 5A). Additionally, two side chains functionalized with a tertiary amine moiety are needed to interact with the grooves: the amine group is crucial for activity since it is protonated at physiological pH, while it disrupts the G4 when substituted with bulky residues (101). The 3,6,9-trisubstituted acridines emerged as the most potent compounds among all the possible regioisomeric series that have been evaluated: they proved to act as G4-mediated telomerase inhibitors. B19 showed telomerase inhibition at nanomolar concentration, with higher affinity for G4 with respect to duplex DNA, and lower cytotoxicity when compared to first generation acridines. It induced long-term growth arrest and replicative senescence in the 21NT breast carcinoma cell line and was the first G4 ligand to prove anticancer activity *in vivo*, against human tumor xenograft models (102,103).

The use of B19 in a viral environment was first analyzed in EBV, to investigate the functional and biochemical characteristics of EBNA1. Results showed that B19 stabilized the viral RNA G4 and, during infection, was able to reduce EBV genome copy numbers in Raji cells. It was also found to induce modest reduction of transcription levels of EBNA2 and EBNA3A and inhibition of EBNA1-dependent DNA replication. These data indicate that G4-interacting molecules can block functions of EBNA1 that are critical for viral DNA replication (71).

**Figure 6.**
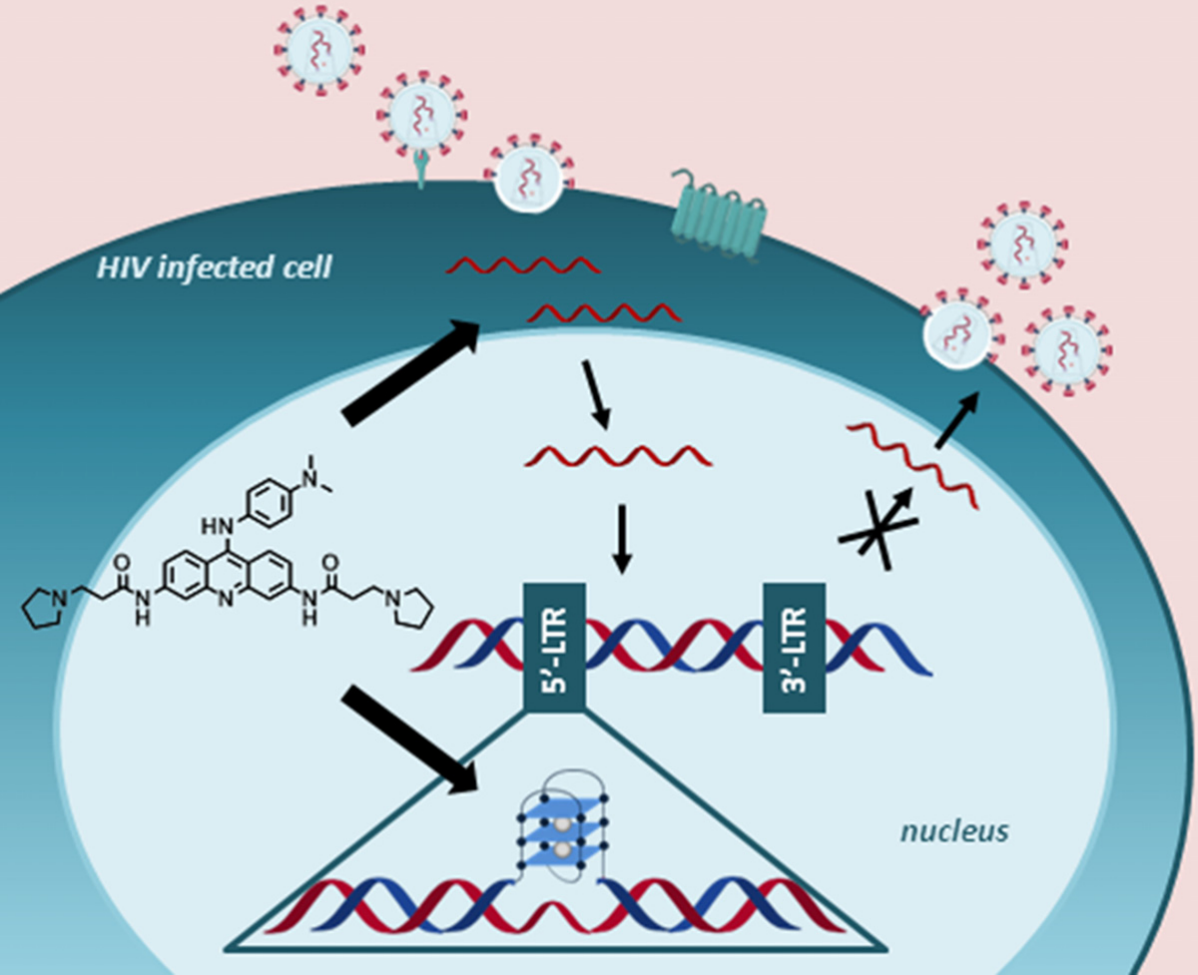
Proposed antiviral mechanism of the G4 ligand B19. In HIV-1 infected cell, B19 exerts a dual mechanism of action: it recognizes and stabilizes the DNA G4s in the 5′-LTR within the proviral genome in the cell nucleus, and it binds to the RNA G4s in the 5′-LTR and 3′-LTR of the viral genome in the cytoplasm: such interactions result in the inhibition of viral direct and reverse transcription.

In the LTR promoter region of the HIV-1 proviral genome, B19 was able to significantly stabilize the naturally occurring G4s, LTR-II and LTR-III, and to induce an additional G4, LTR-IV. In the presence of increasing concentration of B19, LTR promoter activity was decreased of almost 70% with respect to the untreated control, while no activity was detected in a mutated sequence unable to fold into G4s (46). These results confirmed a G4-mediated mechanism of action. The anti-HIV-1 activity of B19 (IC_50_ < 7.9 μM) was tested in various cell lines, against different viral strains and was demonstrated to be G4 mediated. Since G4 structures also formed in the pre-integration viral RNA (55), a dual mode of action both at the pre- and post-integration level was proposed (Figure 6). B19 antiviral activity was tested and confirmed in latent HIV-1 infected cells, where the acridine was able to reduce the viral titer to undetectable level, also in long-term treatment (62).

B19 exerted its G4 stabilizing activity also in the HSV-1 genome, where multiple G4s can form. Treatment with B19 led to a significant antiviral effect (IC_50_ = 8 μM), with reduction in viral DNA synthesis and late proteins production (69).

Moreover, B19 was used in HHV-6A infected cells to evaluate the ability of G4 ligands to impair viral integration in the telomeric region, through stabilization of telomeric G4s. Interestingly, in telomerase expressing cell lines, the frequency of chromosomal integration was reduced up to 50% upon treatment. However, effects of G4 ligands on HHV-6 replication and gene expression are yet to be discovered (81).

Recently, B19 was employed in a luciferase reporter assay to analyze the role of G4s in HBV, where it enhanced promoter activity, suggesting a positive regulatory role of G4s in HBV transcription (85).

Despite its good solubility in aqueous solutions and strong G4 binding, poor permeability across biological barriers, which characterizes most G4 ligands, restrains B19 pharmacological application (104). Nonetheless, B19 is still considered a reference compound in G4 research.

### TMPyP4

The cationic porphyrin compound 5,10,15,20-tetrakis-(*N*-methyl-4-pyridyl)porphine (TMPyP4, **2**, Figure 4) was proposed as G4 binder because of its suitable physical properties, such as molecular size, planar core, positive charges and hydrophobicity, favorable for stacking with the G tetrads (105) (Figure 5B). Biophysical analysis demonstrated that TMPyP4 was actually able to stack and stabilize both parallel and antiparallel G4s, with mild selectivity for quadruplex over duplex DNA (95). Since then, it has been widely employed as a tool to study G4s, especially because of the availability of a negative control compound, TMPyP2 (**3**, Figure 4), which is a structural isomer with *N*-methyl-2-pyridyl residues on the porphine core. Intriguingly, TMPyP2 is sterically hindered from external stacking on the G4 with respect to TMPyP4, producing no biological effects (106,107).

In biological assays, TMPyP4 was shown to inhibit human telomerase (IC_50_ = 6.5 ± 1.4 μM) (108) and downregulate the proto-oncogene *c-myc* expression as well as several *c-myc*-regulated genes containing G4-forming sequences. Such modulation resulted in *in vivo* antitumor activity in different models where the porphyrin was able to decrease tumor growth and prolong survival (109).

In viruses, TMPyP4 was shown to stabilize G4s in the HIV-1 *nef* coding region and to induce their formation within the double-helix conformation. Interestingly, in the TZM-bl reporter cell line, which supports *nef*-dependent HIV-1 replication, the porphyrin inhibited viral infectivity in a dose-dependent manner (48). In addition, TMPyP4 administration was able to block viral replication in two different Jurkat-derived T-cell lines with established HIV-1 latency. Bambara’s research group demonstrated that the antiviral activity was coupled with an increased rate of apoptosis/death when compared to untreated cells, and that this effect was enhanced by association with DNA damage repair inhibitors (62).

In HCV, TMPyP4 was found to stabilize RNA G4s and inhibit HCV C gene expression through a G4-mediated mechanism of action confirmed by an enhanced green fluorescent protein reporter gene system. In addition, in an infectious HCV culture system, administration of the porphyrin led to a dose-dependent decrease of viral RNA levels (89).

TMPyP4 was also employed to investigate the role of G4s in EBOV L gene. It exerted high stabilization of the target G4 RNA in circular dichroism and RNA stop assays. More importantly, after treatment with increasing concentrations of the compound, transcription of the L gene was gradually reduced. To confirm target selectivity, a mutant non-G4-forming sequence was used as a negative control, where TMPyP4 did not produce significant inhibition of transcription. In addition, the porphyrin was found to inhibit replication of EBOV mini-genome, a cell-based approach that uses firefly luciferase as reporter protein and thus can be used as an efficient antiviral screening system (91).

It is worth noting that the low selectivity of TMPyP4 towards G4 structures versus duplex DNA (110) may suggest the antiviral activity to be ascribed to multiple mechanisms of action, limiting its biological and clinical application.

### Perylenes and naphthalene diimides

Perylenes represent a well-known family of G4 ligands, containing a differently substituted, large fused aromatic ring system: they are characterized by a hydrophobic heptacyclic central core, which is responsible for the binding to G quartets through π–π interactions, and by up to four protonated side chains. Accurate SAR studies on this scaffold pointed out two crucial features for G4 binding: the basicity of the system, which prevents the compound from self-aggregation, and the distance between the aromatic central core and the quaternarized nitrogen residue in the side chain, which modulates ligand solubility and affects G4 recognition. The cationic amino moieties in the lateral substituents are thought to regulate specificity for G4 versus duplex DNA (111). PIPER, *N,N’*-bis[2-(1-piperidino)-ethyl]-3,4,9,10-perylenetetracarboxylic diimide (**4**, Figure 4) is the lead compound of this class; it was shown to induce and stabilize G4 structures in telomeres (112), leading to telomere shortening, reduction of cell proliferation and tumorigenicity, and senescence (113).

In the effort to improve the physicochemical properties of the perylene scaffold, progressive surface reduction led to the more promising class of naphthalene diimide (NDI) derivatives. Indeed, it was demonstrated that the dimensions of the planar core modulate the ability of this class of compounds to recognize different DNA conformations. In particular, in the cyclic condensed system at least four rings are required to efficiently target G4s (114). In addition, the NDI planar core can accommodate up to four side chains to enhance G4 affinity. These compounds were found to inhibit telomerase activity in the low micromolar range and to produce short-term cell growth inhibition against MCF-7 and A549 cancer cell lines (115). To improve DNA G4 alkylating properties, further modifications were introduced on the NDI scaffold, which include quinone methides precursors (116,117). These ligands revealed both reversible and irreversible binding properties toward telomeric DNA, with promising duplex versus quadruplex selectivity (118), and were found to impair the growth of different telomerase-positive cancer cell lines following telomerase activity inhibition (119–121). Crystallographic analyses of various NDI–telomere complexes provided a turning point for rational optimization of this class of compounds (122,123). Neidle *et al.* reported that the tested ligands promoted a parallel G4 topology, forming a 1:1 complex with the oligonucleotide. This stoichiometry resulted from the combination of binding site affinity and direct groove interactions that are highly influenced by the protonated moiety in the side chains, which interacts with DNA phosphates in the grooves.

Despite their high molecular weight, NDIs are highly versatile structures, suitable for further medicinal chemistry modifications to improve their pharmacological profile (124,125).

In the antiviral field, PIPER induced and stabilized G4 structures in the *nef* coding region of the HIV-1 genome (48). However, the best results were obtained with core-extended NDI derivatives (c-exNDIs, **5**, Figure 4). This series of compounds, endowed with exceptional solubility properties, has been obtained by fusing the NDI core with a 1,4-dihydroquinoxaline heterocycle. Interestingly, the newly developed ligands displayed greater *in vitro* binding and stabilization activity on viral HIV-1 LTR G4s than the human telomeric sequence, used as a cellular reference G4. Most importantly, the c-exNDIs exhibited very promising antiviral activity in the low nanomolar range (IC_50_ < 25 nM) against different strains of HIV-1, with very low cytotoxicity, yielding a wide and encouraging therapeutic window. The G4-related mechanism of action was proved combining time-related antiviral and reporter assays, using a non-G4-forming LTR-mutant sequence as control. It is reasonable that the higher antiviral activity depends on the selectivity toward the viral G4s, as, during the infection, LTR and telomeric G4s are likely the most abundant species in the cell (61).

The most active c-exNDI was also analyzed in HSV-1 infection. *In vitro* CD and *Taq*-polymerase stop assays indicated that the compound was able to bind and stabilize various G4-forming sequences of the HSV-1 genome. Mass spectrometry competition analysis revealed a stronger preference for HIV-1 G4s over HSV-1, but generally, viral G4s were preferentially bound, when compared to the telomeric G4. Indeed, c-exNDI showed remarkable antiviral activity (IC_50_ = 18.3 ± 1.4 nM). The anti-herpetic effect was ascribed to inhibition of viral DNA replication, as gathered by time-of-addition assay and flow cytometry analysis using acyclovir as reference compound (82). Since c-exNDI selectivity towards HSV-1 G4s *in vitro* resulted to be good but not outstanding, the marked anti-HSV-1 activity was likely due also to the massive presence of viral G4s in the cell nucleus, which was demonstrated to occur during HSV-1 replication (70).

### Pyridostatin

Pyridostatin (PDS, **6**, Figure 4) has been rationally designed on the structural features shared by known G4-binding ligands, as it comprises a potentially planar electron-rich aromatic surface and the ability to participate in hydrogen bonding. Moreover, the rotatable bonds provide a flexibility degree, which makes PDS capable to adapt to the dynamism of G4s. PDS strongly stabilized telomeric G4 with no effect on double-stranded DNA: as a result, the shelterin complex integrity was altered, triggering a DNA-damage response at telomeres (126). Numerous modifications have been introduced in the PDS scaffold to further explore the role of this class in anticancer therapy. Indeed, the obtained analogues showed remarkable growth-inhibitory effects in cancer cell lines and a complete arrest after long-term exposure to the drug. These results emphasize the high potential of these compounds to fine-tune their biological activity (127,128).

In antiviral research, PDS has been used to study the role of G4s in EBV EBNA1 mRNA, where it enhanced the stability of the G4-forming sequence, decreasing EBNA1 synthesis level in a concentration-dependent fashion, both *in vitro* and *in vivo*. As a consequence, EBV-infected cells resulted less efficiently recognized by virus-specific T cells, albeit the mechanism of action still needs to be clarified (75). In HBV, PDS was used to unravel the positive regulatory role of G4s within the preS2/S gene promoter (85).

A PDS analogue, namely PDP (**7**, Figure 4), was employed in HCV G4 research, along with TMPyP4. The PDP-induced stabilization of G4 structures located in the HCV RNA downregulated C gene expression. *In vivo*, PDP inhibited intracellular replication of different HCV genotypes through a confirmed G4-related mechanism of action, resulting in antiviral activity in the low micromolar range (89).

### Bisquinolinium derivatives

Bisquinolinium compounds are characterized by an aromatic nucleus substituted with two protonated quinoline moieties. The first reported compounds present a dicarboxamide-pyridine or -triazine ring as central core: the most promising of these ligands have shown to increase G4 stability in telomeres, with great selectivity over duplex DNA (129). These compounds are able to adopt an intramolecular *syn-syn* H-bond, which was proposed to be critical for G4 recognition, likely because the consequent rigidity of the compound promotes G-quartet overlap. On these bases, the central core was expanded without disrupting the H-bonds, leading to a new disubstituted-1,10-phenanthroline series that displays exceptional selectivity for G4s (130), due to the crescent-like shape which prevents such compounds to intercalate with duplex DNA (Figure 5C) (131,132). PhenDC3 (**8**, Figure 4), the best representative of this class, is a potent telomeric G4 ligand able to reduce telomerase processivity (133).

PhenDC3 was used in KSHV to evaluate its potential role in inhibiting latent viral replication. The ligand was found to elicit a stress response in infected BCBL-1 cells and to stall the replication machinery both in the leading and lagging strands of the KSHV genome. Furthermore, treatment with PhenDC3 resulted in the dramatic reduction (60%) of episome copy number, with no effect on cell growth and proliferation. These data represent the first use of G4 ligands in targeting latent viral infections (77).

PhenDC3 was also used in EBV, where it prevented binding of nucleolin to EBNA1 mRNA G4 and increased the endogenous EBNA1 levels in EBV-infected B cells and in cells derived from a nasopharyngeal carcinoma. These results indicate that the nucleolin–EBNA1 mRNA interaction can also be targeted by antiviral G4-ligands (74).

A summary of G4 ligands and the viruses against which they have been tested is reported in Figure 3.

## DISCUSSION AND FUTURE PERSPECTIVES

In the last decades, research on the role of G4s in the human genome has been quite challenging and promising, leading to the awareness that these high-order structures play key regulatory roles in biological pathways such as transcription, replication, translation and telomere maintenance. The development of G4 binders with encouraging anti-cancer activity has prompted researchers to identify new ways to exploit G4 structures in human diseases, e.g. viral infections.

Because G4s are present both in cell and virus genomes, the challenge in developing antiviral G4 ligands reasonably consists in overcoming selectivity toward viral versus cellular G4s. A major limitation of the so far described G4 ligands is their large flat aromatic core that stacks on the G tetrad, which reduces the chances to discriminate among different G4s. Moreover, they are generally characterized by high-molecular weights and protonated side chains, which are necessary for loops and grooves interaction, but, on the other hand, may affect cellular uptake. Indeed, because of the low selectivity profile and poor drug-like properties, no G4 ligand has advanced beyond Phase II in the drug discovery pathway. Quarfloxin, a fluoroquinolone derivative compound developed by Hurley’s research group (134), is to date the only G4 ligand that has reached Phase II clinical trials but was withdrawn due to bioavailability related problems (35). However, several data presented in the literature indicate that, in general, a certain degree of selectivity is achievable towards the viral G4 of interest in comparison to the telomeric G4, i.e. the most abundant cellular G4 (135). In the case of HIV-1 G4s and c-exNDI compounds, the higher affinity towards the viral structure is likely caused by the extension of the NDI core and thus by the interaction with the viral G4 loop region, which is unique for this G4 (61). In general, loop and groove regions characterize each G4 and thus are amenable for selective recognition. Structural studies on cellular G4/G4 ligand complexes indicated that most G4-binding molecules interact with G4s through quasi-external stacking, in which the heteroaromatic chromophore of the small molecules is π–π stacked onto the face of an external G-quartet (136) (Figure 5) and onto the side chains positioned in the G4 grooves (94). It is therefore conceivable that the reported antiviral activity of G4 ligands is mediated by an increased interaction, hence affinity, with the groove/loop moiety of the viral G4s. To date, only one viral G4 structure has been resolved through NMR spectroscopy (49), therefore future NMR and crystallographic resolutions of viral G4s and G4/G4 ligand complexes are necessary to define the viral G4s architecture. This could help researchers identifying possible unique G4 structures which could lead to the design and development of selective molecules.

In other cases, G4 ligands did not show significant selectivity for the viral versus telomeric G4s, and the G4s present in oncogene promoters were usually strongly bound by the tested compounds (82). Nonetheless, the data so far presented on the antiviral use of G4 ligands have shown in general very promising activity against a wide range of virus species. One possible explanation is that the amount of the viral G4s in the infected cells largely surpasses that of the cellular G4s (70). Indeed, usually cells are exploited to function as factories in the production of new viral genomes that are eventually assembled into new mature virus particles (See Figure 2 for the viral infection cycle). It is thus conceivable that the viral G4s become largely more abundant than the cellular G4s during virus replication. At least in one case this eventuality has been demonstrated: in HSV-1 there is a sharp increase in the number of viral G4s during viral DNA replication (70). Combining the abundance of G4s per genome and the number of new genomes, the amount of viral G4s could outstand that of cellular G4s by several logs per cell. In addition, the so far identified viral G4s are usually key regulatory elements of the virus life cycle and their stabilization/unfolding by G4 ligands can likely explain the resulting massive virus inhibition. If this behaviour is demonstrated also in other viruses, it would be possible to exploit G4 ligands that are not strictly selective for the viral G4s. This scenario would highly and rapidly expand the research and pharmacological application of G4 ligands as antiviral agents.

A further point to be addressed is the necessity to standardize methods to study the antiviral activity of the G4 ligands. One starting point should be the detection of the inhibitory activity of the ligand on the virus life cycle. If an effect is obtained, further investigation on the mechanism of action has to be performed. In this regard, the time of addition method (137) can be of assistance as it indicates the last viral step at which the compound is active and it thus narrows the possible molecular targets. However, because of the complexity and uniqueness of each virus, the investigation of the target and mechanism of action at the molecular level may not be straightforward. For example, PDS inhibited EBNA1 synthesis *in vitro* but not in cells, while PhenDC3 in cells led to the exact opposite effect, i.e. enhanced EBNA1 synthesis (74,75). It is likely that multiple G4-mediated mechanisms are involved in the observed outcomes.

Finally, targeting G4s in the viral genomes leads to the exciting possibility of affecting viruses that undergo latency. These viruses, such as HIV, the herpes and papilloma virus families, comprise an initial acute infection and a subsequent latent infection. The latter is characterized by the maintenance of the virus genome in the human host for the entire life of the host. The latent virus may reactivate from time to time to produce new mature virus. Current therapies that normally target viral proteins fail to remove the latent virus, i.e. the virus genome, from its host. Selectively targeting the viral genome in a G4-mediated approach would allow removing not only the replicating virus but also the latent one, therefore eradicating so far incurable infective agents.

In this picture it is worth considering the virus-induced manipulation of host chromatin. In recent years, studies about the role of chromatin in viral infections showed dynamic virus–host chromatin interactions and chromatin machinery modulation by virus encoded proteins (138). For example, the HSV-1 epigenetic regulation of viral chromatin by viral gene products plays a key role in determining whether the virus develops a lytic or latent infection (139). Considering the recent evidences reported by Hänsel-Hertsch *et al.* that G4 formation reflects the suppressive role of heterochromatin and that it occurs only in highly transcribed regulatory nucleosome-depleted chromatin regions (13), it would be interesting to understand how the virus and its G4s affect and could be affected by such a complex mechanism.

To conclude, all the data reported in this review indicate that: i) G4 structures are crucial elements in the regulation of viruses’ life cycle, both in lytic and latent states; ii) G4 ligands efficiently act as antiviral agents. This should encourage researchers to continue investigating on G4-binding small molecules: as a matter of fact, albeit quarfloxin clinical evaluation did not progress, its success in Phase I clinical trial, i.e. optimal toxicity profile (35), suggests that improvements of G4 ligand pharmacological profiles will very likely lead to concrete clinical applications of these compounds.

Therefore, research in the next future will need to improve i) the understanding of G4 activity and regulation at the viral level, ii) the selectivity of G4 ligands toward the viral versus cellular G4s, iii) the drug-like properties of the antiviral G4 ligands to be employed in *in vivo* studies.

G4-mediated antiviral drugs may represent a significant turning point in the management of viral infections, especially for people who cannot access immunization, like immunocompromised patients or elderly people. In addition, the G4-mediated antiviral effects reported in latent infections (62) may pave the way for cutting-edge therapeutic approaches in the treatment of human fatal malignancies related to latent viruses, such as AIDS, herpes- and HPV-related cancer.
